# Metabolic and Transcriptomic-Based Characterization of *Streptococcus salivarius* ssp. *thermophilus* Snew Fermentation in Milk

**DOI:** 10.3390/foods14030530

**Published:** 2025-02-06

**Authors:** Ye Wang, Haijie Zhao, Huilin Zhang, Baochao Hou, Weilian Hung, Jian He, Chao Liang, Baolei Li, Chaoxin Man, Yujun Jiang, Yu Zhang, Ling Guo

**Affiliations:** 1 Key Laboratory of Dairy Science, College of Food Science, Northeast Agricultural University, Harbin 150030, China; wyyyee2000@163.com (Y.W.);; 2 National Center of Technology Innovation for Dairy, Hohhot 010000, China; 3 Food Laboratory of Zhongyuan, Luohe 462300, China

**Keywords:** *Streptococcus salivarius* ssp. *thermophilus*, metabolomics, transcriptomics, fermented milk, biochemical characteristics

## Abstract

Fermented milk has a long history. It is fermented by lactic acid bacteria and is rich in protein, minerals, vitamins, and other nutrients. As people’s pursuit of quality of life improves, consumers are paying increasing attention to fermented milk. *Streptococcus salivarius* ssp. *thermophilus* is commonly used to make fermented milk. This study investigated the fermentation characteristics and physicochemical properties of *Streptococcus salivarius* ssp. *thermophilus* Snew-fermented milk, as well as transcriptomic and metabolomic analyses of different fermentation stages. *Streptococcus salivarius* ssp. *thermophilus* Snew can be used as a fermenter strain, as evaluated from the point of view of fermentation time, titratable acidity, post-acidification, viable bacteria count, water holding capacity, and viscosity. The flavor and odor of Snew-fermented milk varied across fermentation stages. The analysis of the detected volatiles revealed that ketones and esters were the main substances responsible for the flavor of Snew-fermented milk. The differentially expressed genes and differential metabolites screened from several categories, such as carbohydrates, proteins, amino acids, fats, and fatty acids, varied at different fermentation stages, while differentially expressed genes and differential metabolites were also threaded together for joint analysis in this study. This study provides theoretical guidance for the practical production application of *Streptococcus salivarius* ssp. *thermophilus* in cow’s milk fermentation.

## 1. Introduction

Milk is widely consumed for its nutritional value and accessibility. Fermented milk, with its distinctive flavor and abundance of nutritional benefits, has gradually gained popularity with the advancement of society. Fermented milk production has evolved from traditional autonomous fermentation methods to modern industrialized and standardized procedures. One of the greatest characteristics of fermented milk is its unique sourness and flavor, which is mainly determined by the lactic acid produced by lactic acid bacteria during the fermentation process. Different species of lactic acid bacteria and their fermentation characteristics have a significant impact on the final taste, texture, and nutritional composition of fermented milk [1]. In addition, the health benefits of fermented milk, such as the improvement of the gut microbiota and enhancement of immunity, are among their distinguishing features, which have made fermented milk a widely consumed functional food worldwide [2]. The definition of fermented milk by the Food and Agriculture Organization of the United Nations and the World Health Organization, according to the International Codex Alimentarius Standard, is a dairy product obtained after milk fermentation [3].

*Streptococcus salivarius* ssp. *thermophilus* is a non-pathogenic Gram-positive bacterium belonging to the phylum *Firmicutes* and family *Streptococcaceae* [4]. *S. thermophilus* has been widely used in fermented milk. Dan et al. [5] showed the excellent quality of milk fermented with *S. thermophilus* MGA45-4 during storage. Li et al. [6] demonstrated that *S. thermophilus* S10 contributes to the distinctive flavor, taste, and aroma of the finished fermented milk product. Ge et al. [7] also explored the role of *S. thermophilus* CICC 6038 and *Lactobacillus terreus Bulgaria* CICC 6047, suggesting an important role for *S. thermophilus* in fermentation. Existing studies on *S. thermophilus* fermented milk have focused on the physicochemical characteristics of the fermentation process alone or in combination, the changes occurring in the fermented milk end products, and in the storage of the finished product. The gene expression and metabolites of *S. thermophilus* in the fermentation process have not been studied and analyzed.

The application of histological techniques in fermented food research provides an understanding of the metabolism and function of microbial communities and their impact on fermented products [8]. Transcriptomics has been used by researchers to explore *S. thermophilus’* ability to utilize galactose and extracellular polysaccharide biosynthesis, or to focus on the analysis of a specific gene [9,10,11,12,13], as well as to reveal the overall genetic changes in *S. thermophilus* during a pH-controlled batch fermentation process [14]. Using metabolic analysis, Xie et al. [15] showed that the synthesis and metabolism of a number of these non-volatile and volatile compounds are closely related to the fermentation properties of *S. thermophilus* in fermented food. Studies have found that different strain treatments, fermentation conditions, and fermentation stages have different effects on metabolic pathways and metabolites through histological techniques—for example, the galactose metabolic pathway, ketone flavor substances, and functional biomolecules [16,17,18]. However, previous studies have been based on the growth of *S. thermophilus* in a normal medium, and there is a lack of biochemical characterizations of *S. thermophilus* at different stages of growth in the cow’s milk matrix. At the same time, most studies have focused on either the transcriptomics or metabolomics of *S. thermophilus* alone and have not yet analyzed the two groups jointly.

Therefore, in this study, the three stages of *S. thermophilus* Snew fermentation in cow’s milk were comparatively analyzed to reveal the differences in physicochemical and sensory properties and volatile characteristics of *S. thermophilus* in different fermentation stages. In addition, this study is the first to perform metabolite and gene integration analyses covering multiple categories such as carbohydrates, proteins, amino acids, fats, and fatty acids. These novel analyses provide the basis for an in-depth understanding of the biochemical properties of *S. thermophilus* during fermentation and help to further assess its viability as a fermentation strain. The present study provides a theoretical basis for the various changes produced by *S. thermophilus* in bovine milk fermentation.

## 2. Materials and Methods

### 2.1. Materials and Cell Lines

*S. thermophilus* Snew was isolated in the laboratory from traditional fermented dairy products in Inner Mongolia. Skimmed milk powder was purchased from Yili, China. Sucrose, sodium hydroxide, and sodium chloride (analytically pure) were purchased from Tianjin Tianli Chemical Reagent Co., Tianjin, China. M17 solid medium was purchased from Qingdao Haibo Biological Co., Qingdao, China.

### 2.2. Preparation of Fermented Milk

The method of Wang et al. [19] was referred to and modified for the preparation of fermented milk samples. Weighed 12 ± 0.1 g of skimmed milk powder was dissolved in 100 mL of distilled water and autoclaved at 105 °C for 15 min. After the temperature was reduced to 50 °C, 8 % sucrose was added and mixed using a flapping homogenizer. *S. thermophilus* Snew was added to the homogenized skimmed milk medium at an inoculum of 5 × 10^7^ CFU/mL, and fermentation was carried out at 37 °C. A pH value of 4.5 is usually considered the end point of fermented milk [20], so we set the late pH at 4.5. A fermentation broth with different pH levels was used as a sampling point to reveal the overall gene changes of *S. thermophilus* at different pH levels during fermentation in the normal medium. The researchers chose four phases: a lagging phase (2 h of fermentation), exponential mid phase (5 h of fermentation), exponential late phase (7 h of fermentation), and stabilization [14], with the first three phase times corresponding to the time when the pH reached 6.0, 5.1, and 4.5, respectively. Some studies also chose time points for fermentation initiation, fermentation curd (pH 5.20), fermentation termination (F2 4.60), or other similar pH levels [21,22]. We chose pH values of 6.0 ± 0.01 (Group Snew-1), 5.1 ± 0.01 (Group Snew-2), 4.5 ± 0.01 (Group Snew-3) for the pre, mid, and post phases.

#### 2.2.1. Determination of pH and Titratable Acidity

A pH meter (Seven Compact™ S210, Mettler toledo, Zurich, Switzerland) with an ISM^®^ electrode was used to monitor the pH changes in the fermented milk, and 3 mL samples were taken every hour to determine the pH value until a pH value of 4.5 was reached, at which point the fermentation was considered complete as the end point of fermentation. Before the measurement of pH, the pH meter was calibrated using standard buffer solutions with pH levels of 4.01, 7.00, and 10.01. Standard buffer solutions with a pH level of 10.01 were used to calibrate the pH meter. After the calibration was completed, the electrode was cleaned thoroughly and wiped dry, and then the pH electrode was carefully immersed in the sample to be measured, making sure that the probe of the electrode was fully immersed in the liquid. When pH meter showed a stable reading, the data were recorded.

Hourly, 10 mL samples were taken for the determination of titratable acidity and diluted and homogenized with 20 mL of water (boiled for 15 min and cooled for use). The acidity of the fermented milk was calculated by titrating the diluted sample with 0.10 mol/L NaOH standard solution until a pH level of 8.3 was reached [23]. The titratable acidity (TA) was expressed in Gilner degrees (°T). The calculation formula is as follows:

TA (°T)=V × 10

V denotes the volume of the NaOH standard solution utilized.

#### 2.2.2. Determination of Post-Acidification

The fermented samples were stored at 4 °C, and their titratable acidity was measured after 1, 3, 10, and 15 days of storage. The determination method used is described above.

#### 2.2.3. Determination of Viable Bacteria Count

The viable bacteria count was determined by an in-base method [24]. The samples (1 mL) of unfermented (Snew-0), pre, mid, and late fermentation were collected and diluted with 85% sterilized saline to the preferred dilution. Then, the diluted samples were placed into a petri dish on top of 15–20 mL of M17 agar solid medium at 45 °C. The petri dishes were then incubated for 48 h at 37 °C. The 30–300 colonies present in the dish were measured and recorded as Log CFU/mL.

#### 2.2.4. Determination of Water Holding Capacity (WHC)

The method of Grasso et al. [25] was adopted with modifications. A 50 mL centrifuge tube was selected for weighing, and the unfermented (Snew-0), pre-, mid-, and late-fermentation samples (10 g each) were placed in the tubes and centrifuged at 5500 rpm for 30 min (SCILOGEX CF1524R, Rocky Hill, CT, USA). After removing the supernatant, the tubes were inverted for 10 min to ensure complete draining of the supernatant before measuring the mass of the precipitate. The calculation formula is as follows:

$$\mathrm{WHC}\;\%=\left[(W2-W0)\;⁄\;(W1-W0)\right]\;\times \;100\;\%$$

W0: weight of empty centrifuge tube; W1: centrifuge tube and sample weight; W2: weight of centrifuge tube and sample after centrifugation.

#### 2.2.5. Determination of Viscosity

The viscosity of fermented milk was measured at 100 rpm using a DVS+ viscometer (Brookfield, WA, USA) equipped with a rotor of LV-4 (64), and values were recorded at 30 s intervals. Each group was measured thrice.

### 2.3. E-Nose and E-Tongue

E-tongue and e-nose [26] determination methods were used. Samples from the pre-, mid-, and post-fermentation stages were loaded into headspace injection bottles and analyzed using an electronic nose system (PEN 3, AIRSENSE, Schwerin, Germany), with a 60 s cleaning of the sensor and a 60 s determination time (1 s interval). Three measurements were taken. The samples were then homogenized and measured using an electronic tongue system (SA402B, INSENT Corporation, Atsugi-shi, Kanagawa-ken, Japan) after soaking and cleaning the sensor probe. Each sample was measured three times in a space cup.

### 2.4. Determination of Volatile Substances

The method for determining volatile substances is based on Yang [27], with minor modifications. The samples of 3 mL from the pre-, mid-, and post-fermentation stages were loaded into a headspace injection vial, an aged extraction needle was inserted, and the samples were extracted in a water bath at 55 °C for 60 min and then inserted into a gas chromatography–mass spectrometry coupled machine (GCMS-QP2020 NX, Shimadzu, Japan) for subsequent experiments. (1) Chromatographic conditions: the carrier gas is He and the flow rate is 1.0 mL/min; there is no split injection, and the injection port temperature is 250 °C. Programmed heating mode: the starting temperature is 35 °C, and is held for 5 min; then, it is increased to 140 °C at the rate of 5 °C/min, held for 2 min, increased to 250 °C at the rate of 10 °C/min, and held for 3 min. (2) Mass spectrometry conditions: full-scan mode; EI ion source; electron energy of 70 eV; temperature of the ion source is 230 °C; mass scanning range of m/z 35~500 U; emission current 100 µA, detection voltage 1.4 kV, no solvent delay. (3) Solid-phase microextraction (SPME) extraction conditions: equilibrium at 55 °C for 60 min. (4) desorption conditions: desorption at 250 °C for 3 min. (5) Column: DB-5MS.

### 2.5. Transcriptome Sequencing

RNA degradation and contamination were monitored on 1% agarose gels. RNA integrity was assessed using the RNA Nano 6000 Assay Kit of the Bioanalyzer 2100 system (Agilent Technologies, Santa Clara, CA, USA).

Total RNA was used as input material for the RNA sample preparations. mRNA was purified from total RNA using probes to remove rRNA. Fragmentation was performed using divalent cations under elevated temperatures in the first-strand synthesis reaction buffer (5×). First-strand cDNA was synthesized using the random hexamer primer and M-MuLV Reverse Transcriptase, and then RNase H was used to degrade the RNA. In the DNA polymerase I system, dUTP is used to replace the dNTP of dTTP as the raw material to synthesize the second strand of cDNA. The remaining overhangs were converted into blunt ends via exonuclease/polymerase activities. After the adenylation of 3’ ends of DNA fragments, an adaptor with a hairpin loop structure was ligated to prepare for hybridization. Then, the USER Enzyme was used to degrade the second strand of cDNA containing U. In order to select cDNA fragments of preferentially 370~420 bp in length, the library fragments were purified with the AMPure XP system (Beckman Coulter, Beverly, MA, USA). Then, PCR was performed with Phusion High-Fidelity DNA polymerase, Universal PCR primers, and Index Primer. Finally, PCR products were purified (AMPure XP system), and library quality was assessed using the Agilent Bioanalyzer 2100 system. The clusterProfiler software was used for the Gene Ontology (GO) functional enrichment analysis (https://geneontology.org/, accessed on 24 March 2023) of differential gene sets and KEGG pathway enrichment analysis (https://www.genome.jp/kegg/pathway.html, accessed on 1 April 2023).

### 2.6. Metabolomics

The sample (100 μL) was taken and mixed with 400 μL of extraction solution (MeOH: ACN, 1:1 (*v*/*v*)); the extraction solution contained deuterated internal standards, and the mixed solution was vortexed for 30 s, sonicated for 10 min in a 4 °C water bath, and incubated for 1 h at −40 °C to precipitate proteins. Then, the samples were centrifuged at 12,000 rpm (RCF = 13,800× *g*), R = 8.6 cm) for 15 min at 4 °C. The supernatant was transferred to a fresh glass vial for analysis. The quality control (QC) sample was prepared by mixing an equal aliquot of the supernatant of samples.

The raw data were converted to mzXML format using Protein Wizard (http://www.proteowizard.org/, accessed on 1 June 2023). The data were developed using R and based on XCMS (https://xcmsonline.scripps.edu/, accessed on 15 June 2023.) for peak detection, extraction, alignment, and integration. R package and BiotreeDB (v3.0) were used for metabolite identification.

### 2.7. Statistical Analysis

All experiments were performed in triplicate, and the results are expressed as mean ± standard deviation (SD) (*n* = 3). Statistical differences were analyzed by one-way Analysis of Variance (ANOVA), and statistically significant differences were calculated using the SPSS software ver. 19.0 (IBM, Armonk, NY, USA). Values of *p* < 0.05 were considered significantly different.

## 3. Results

### 3.1. Physicochemical Indicators of Fermented Milk

#### 3.1.1. Fermentation Time and Acidity

**Figure 1:**
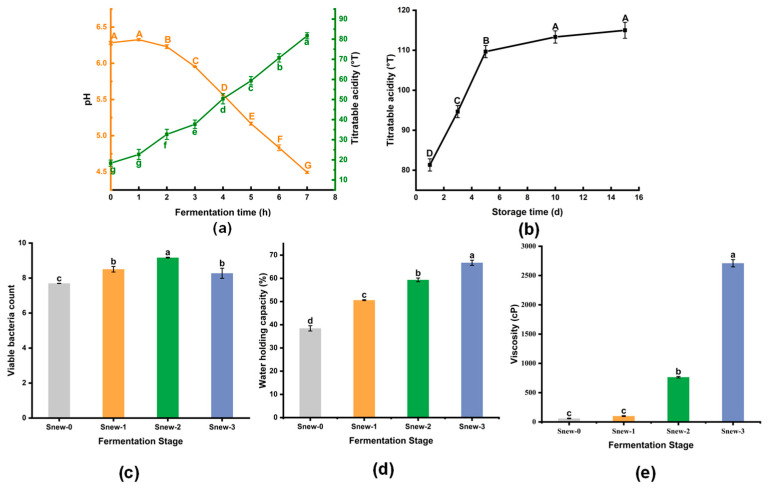
Changes in the physicochemical properties of Snew-fermented milks. (**a**) Fermentation time and titratable acidity. (**b**) Post-acidification. (**c**) Viable bacteria count. (**d**) Water holding capacity. (**e**) Viscosity. Different letters represent significant differences. Capital letters and lowercase letters represent different measurement indicators respectively.

LAB produces lactic acid as it grows, and the accumulation of lactic acid leads to a decrease in milk pH [28]. After milk is injected with *S. thermophilus* Snew, the pH of the milk reaches 4.5 in 7 h. (Figure 1a). The pH decreases gradually over the first 2 h, then more rapidly, stabilizing as fermentation progresses. Notably, all Snew-fermented milks had a rise in pH at the beginning of fermentation, possibly due to the excess nitrogen in the fermentation substrate, release of amino nitrogen, or production of physiological alkaline substances [29]. Milk showed a gradual increase in acidity during Snew fermentation (Figure 1a). The acidity after fermentation was at 81.7 ± 1.53 °T.

#### 3.1.2. Post-Acidification

As shown in Figure 1b, the titratable acidity of *S. thermophilus* Snew-fermented milk increased rapidly with a rate of change of 14.5 °T/d within 5 days of storage at 4 °C, and the acidity of the fermented milk changed more gently during 6–15 days of storage. After 15 days of storage, the acidity of fermented milk was 115.0 ± 2.00 °T.

#### 3.1.3. Viable Bacteria Count, WHC, and Viscosity

During the fermentation process, the viable bacteria count of the fermented milk showed an increasing and then decreasing trend (Figure 1c), and the total viable bacteria count in Snew-2 was 9.2 ± 0.02 Log CFU/mL, which was significantly higher than that of the other two stages (*p* < 0.05). The reason for this is that the abundance of nutrients, such as reducing sugars in the milk during the pre-fermentation period, provides the primary source of energy for *S. thermophilus* [30], but the gradual reduction in nutrients, and the decreasing pH as the fermentation progressed, limit the survival of *S. thermophilus*.

The WHC refers to the ability of fermented milk to retain all or part of its water; a low WHC of fermented milk will lead to whey separation, resulting in a poor fermented milk quality [31]. LAB promotes the binding of casein micelles, increasing the viscosity and WHC of the milk during fermentation [32]. It also promotes the production of exopolysaccharides, improving fermented milk’s texture and organoleptic characteristics. As these changes occur, the form of the fermented milk gradually changes from liquid to solid [33]. As a result, the viscosity and WHC of the Snew-fermented milk gradually increased (Figure 1d,e).

#### 3.1.4. Analysis of Odor and Taste

**Figure 2:**
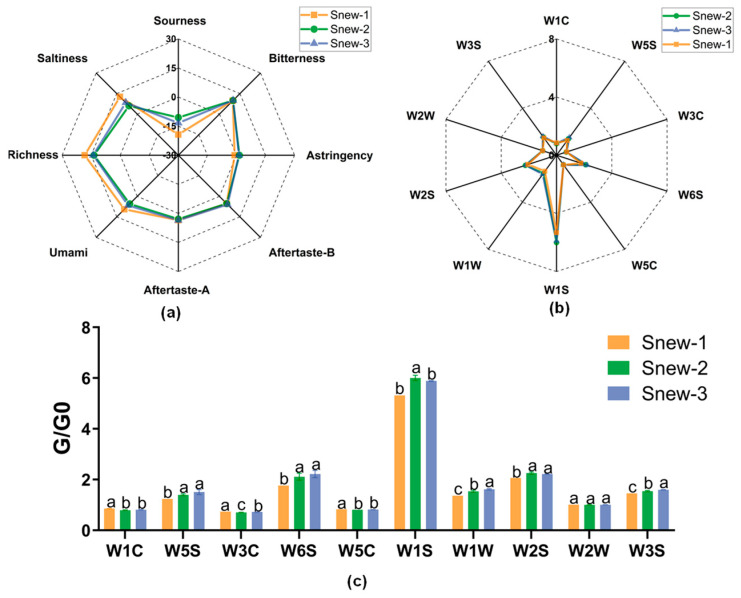
Snew-fermented milk changes in odor and taste. (**a**) Changes in flavor of fermented milk in different groups. (**b**) Changes in the odor of fermented milk in different groups. (**c**) Changes in G/G0 values of fermented milk odor in different groups. G/G0 represents the ratio of the sensor signal. G represents the signal response when the gas is activated on the sensor, and G0 represents the initial signal response of the sensor in the reference state (pure air after cleaning). (Sensors W1C, W5S, W3C, W6S, W5C, W1S, W1W, W2S, W2W, and W3S detected substances as aromatic compounds, nitrogen oxides, ammonia and aromatic compounds, hydrides, olefinic compounds, alkanes, sulfur compounds, alcohol compounds, aromatic compounds (containing sulfur), alkanes, and aliphatic compounds). Different lowercase letters represent significant differences.

As the fermentation progressed, the sourness and astringency values of Snew-fermented milk increased, while the saltness, richness, and umami showed varying degrees of decrease. The changes in flavor of Snew-fermented milk occurred mainly in the Snew-1 group, with lesser differences in the Snew-2 and Snew-3 groups (Figure 2a).

Compared with the Snew-1 group (Figure 2b,c), the sensor detected a significant increase in the levels of nitrogen oxides, hydrides, alkanes, sulfur compounds, alcohol compounds, and alkanes, and aliphatic compounds in fermented milk in the Snew-2 group levels increased significantly (*p* < 0.05). The levels of aromatic compounds, ammonia and aromatic compounds, and olefinic compounds decreased. Compared with the Snew-2 group, the sensor detected an increase in the levels of ammonia and aromatic compounds and olefinic compounds and a decrease in the levels of alkanes in the Snew-3 group, and the same trend was observed for the other groups. Aromatic compounds (containing sulfur) did not change significantly throughout the fermentation stage.

### 3.2. Volatile Substances

**Figure 3:**
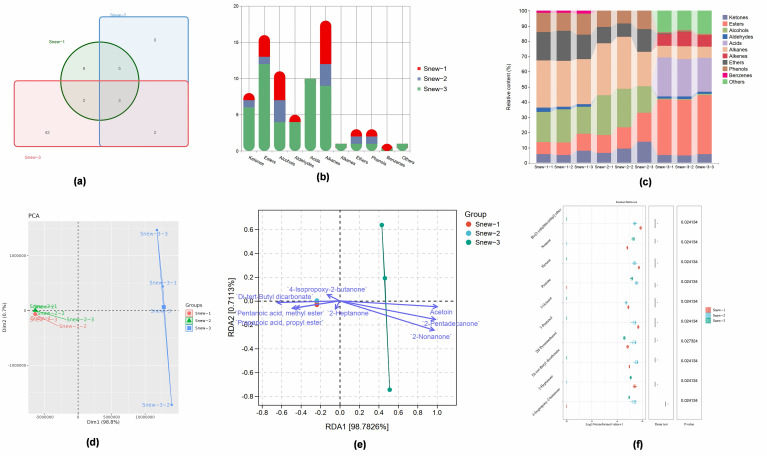
Changes in volatile metabolites in different groups. (**a**) Number of volatile metabolites in different groups. (**b**) Volatile metabolite species in different groups. (**c**) Relative abundance of volatile metabolites. (**d**) Principal component analysis (PCA). (**e**) Redundant analysis of RDA in different groups. (**f**) Differences in shared metabolites.

As shown in Figure 3a, a total of 62 volatile metabolites were detected during the fermentation period, and 18, 10, and 49 volatile metabolites were detected in Snew-1, Snew-2, and Snew-3, respectively. There were 8, 0, and 42 volatile metabolites in Snew-1, Snew-2, and Snew-3 that were not identical to the other groups, respectively, thus indicating that the production of volatile substances increased with fermentation, which conferred a rich flavor profile to the fermented milk. In terms of volatile metabolite species (Figure 3b), among acids and other substances, squalane is the only one present in Snew-3, indicating that such substances are mainly concentrated in the late fermentation stage where they are formed and enriched with ketones, esters, alcohols, aldehydes, alkanes, and hydrocarbons. In addition, in terms of the relative abundance of various volatile metabolites (Figure 3c), it was found that alkane substances had higher relative abundance in Snew-1 and Snew-2, and there was no significant difference between the two. Ketones and alcohols had the highest relative abundance in Snew-2. Esters, acids, and olefins had the highest relative abundance in Snew-3, indicating that the alkane substances decreased with the progression of the fermentation and that ketones and alcohols were produced in large quantities in the middle stage of fermentation. Esters, acids, and olefinic substances were produced in large quantities in the late fermentation stage. According to the principal component analysis (PCA) (Figure 3d), Snew was not significantly differentiated from Snew-1 and Snew-2 intergroup samples but was significantly differentiated from Snew-3 among the three stages during fermentation, indicating that the changes in volatile substances during the fermentation of Snew-fermented milk were mainly concentrated in the later stages of fermentation. In RDA redundancy analyses (Figure 3e), 2-Heptanone, 4-Isopropoxy-2-butanone, Di-tert-Butyl dicarbonate, Pentanoic acid, methyl ester, Propanoic acid, and propyl ester were mainly indicated in Snew-1 and Snew-2, and Acetoin, 2-Pentadecanone, and 2-Nonanone were mainly indicated in Snew-3, suggesting that ketones and esters are the main flavor substance classes in Snew-fermented milk.

By analyzing twelve metabolites common to two or three phases, there was no overall significant difference between groups for two volatile substances and an overall significant difference between groups for ten volatile substances (Figure 3f). There was no overall significant difference between groups for two volatiles and an overall significant difference between groups for ten volatile substances (Figure 3f).

### 3.3. Transcriptomics Analysis

#### 3.3.1. RNA-Seq Sequencing and Sample Analysis

**Figure 4:**
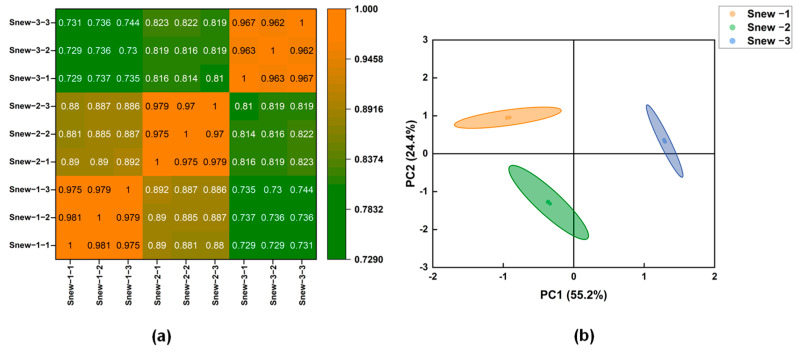
(**a**) Correlation plot of gene expression in different groups. (**b**) Plot of PCA of gene expression in different groups.

The constructed cDNA libraries were up-sequenced using the Illumina platform to obtain raw down-sequencing data and further filtered. The samples were down-sequenced to obtain 6,552,236~8,993,910 raw reads, and 6,428,384~8,912,848 clean reads were obtained after clipping and removing low-quality data. The number of filtered bases was 1.0–1.2 Gb. Q20% indicates the percentage of bases with a base identification accuracy of 99% or more, and Q30% indicates the percentage of bases with a base identification accuracy of 99.9% or more. The results show that Q20% is greater than 96% and Q30% is greater than 90% (Table S1), indicating high sequencing quality. These results can be used for subsequent analysis. The heat map analysis showed that the correlation coefficients of all three groups were greater than 0.7 (Figure 4a). The PCA showed (Figure 4b) that the samples were clustered within the groups and dispersed between the groups.

#### 3.3.2. Differentially Expressed Genes (DEGs)

**Figure 5:**
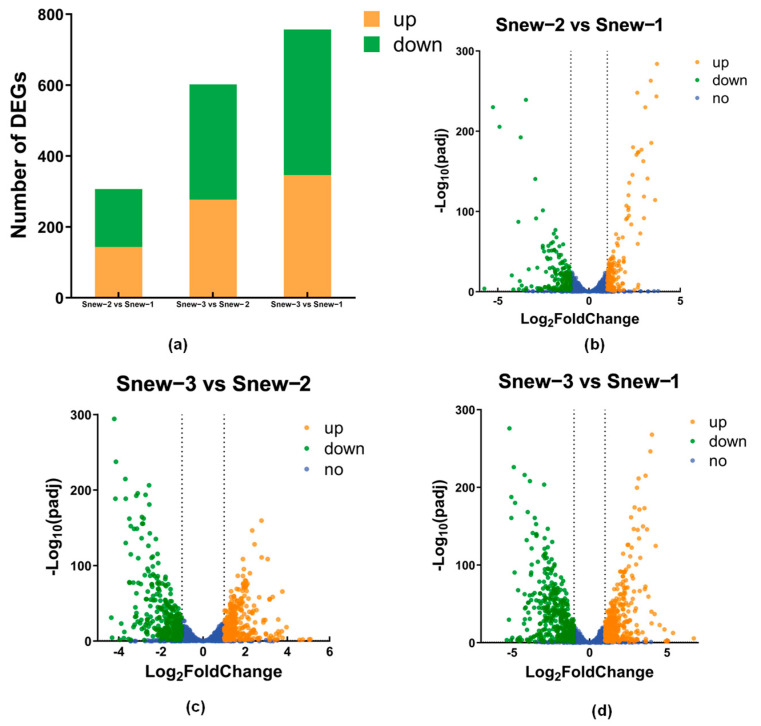
(**a**) Up- and down-regulation of significantly differentially expressed genes (DEGs) in different groups. (**b**–**d**) Volcano plot of significantly differentially expressed genes in different groups.

Based on the screening criteria of |log 2 (Fold Change)| > 0 and padj < 0.05, the samples were screened for genes with significantly different expression levels in different states. The results showed (Figure 5) that 307 (143 up and 164 down), 602 (277 up and 325 down), and 757 (346 up and 411 down) were detected in the three groups, respectively. It can be seen that fewer fermented milk differential genes were detected in Snew-2 vs. Snew-1, and the differences caused by the strains gradually increased as fermentation continued, so that there were significantly more DEGs, up-regulated genes, and down-regulated genes in the Snew-3 vs. Snew-2 and Snew-3 vs. Snew-1 groups than in the Snew-2 vs. Snew-1 group.

As can be seen from Table 1, in carbohydrate metabolism, the genes with significantly up-regulated expression in the Snew-2 vs. Snew-1 group were *malQ* (4-alpha-glucanotransferase), *galT* (UDP-glucose-hexose-1-phosphate), and *pfkB* (1-phosphofructokinase); in the Snew-3 vs. Snew-2 group, the genes with significantly up-regulated expression were *manA* (mannose-6-phosphate isomerase) and *pfkB*. And in the Snew-3 vs. Snew-1 group, the genes with significantly up-regulated expression were *malQ*, *galT*, and *pfkB*. Up-regulated genes were *malQ*, *galT*, *galE* (UDP-glucose 4-epimerase GalE), *pfkB*, *galM* (galactose-1-epimerase), and *manA*. By analyzing the significant DEGs, Snew was found to focus on glycolysis, fructose, glucose, mannose, and lactose metabolism in carbohydrate metabolism. Moreover, the expression of genes encoding polysaccharide synthesis was found to be significantly up-regulated during fermentation. This result is consistent with the trend of texture formation in fermented milk.

In protein and amino acid metabolism, the gene expression of peptidase *pepC* was significantly up-regulated in the Snew-2 vs. Snew-1 group; in arginine anabolism, the gene expression of *argB*, *argJ*, *argC*, *argF*, and *argH* was significantly down-regulated; and in tryptophan metabolism, the gene expression of *trpA* and *trpB* was significantly down-regulated. Regarding the Snew-3 vs. Snew-2 group, in serine and threonine metabolism, *hprK* gene expression was significantly up-regulated, and *thrB*, *serB*, and *serC* gene expression was significantly down-regulated; the gene expression of *trpA* and *trpB* was significantly down-regulated. In glutamate metabolism, gene expression of *gdhA* was significantly down-regulated. In the Snew-3 vs. Snew-1 group, the gene expression of the peptidase *pepA* was significantly up-regulated; the gene expression of *argJ*, *argC*, *argF*, and *argH* was significantly down-regulated; the gene expression of *gdhA* was significantly down-regulated; the gene expression of *hprK* was significantly up-regulated; and the gene expression of *thrB*, *serB*, and *serC* was significantly down-regulated (Table 2). It was found that Snew protein hydrolysis and arginine metabolism were mainly concentrated in the pre-fermentation period, serine and threonine metabolism and glutamate metabolism were mainly concentrated in the late fermentation period, and tryptophan metabolism occurred throughout the fermentation.

In fat and fatty acid metabolism, *trpC* (indole-3-glycerol phosphate synthase TrpC) gene expression was significantly down-regulated in the Snew-2 vs. Snew-1 group; *gap* (type I glyceraldehyde-3-phosphate) gene expression was significantly up-regulated in the Snew-3 vs. Snew-2 group; and *gap* gene expression was significantly up-regulated in the Snew-3 vs. Snew-1 group (Table 3). The fat and fatty acid metabolism of Snew was found to be concentrated throughout the fermentation process.

#### 3.3.3. KEGG Enrichment Analysis

**Figure 6:**
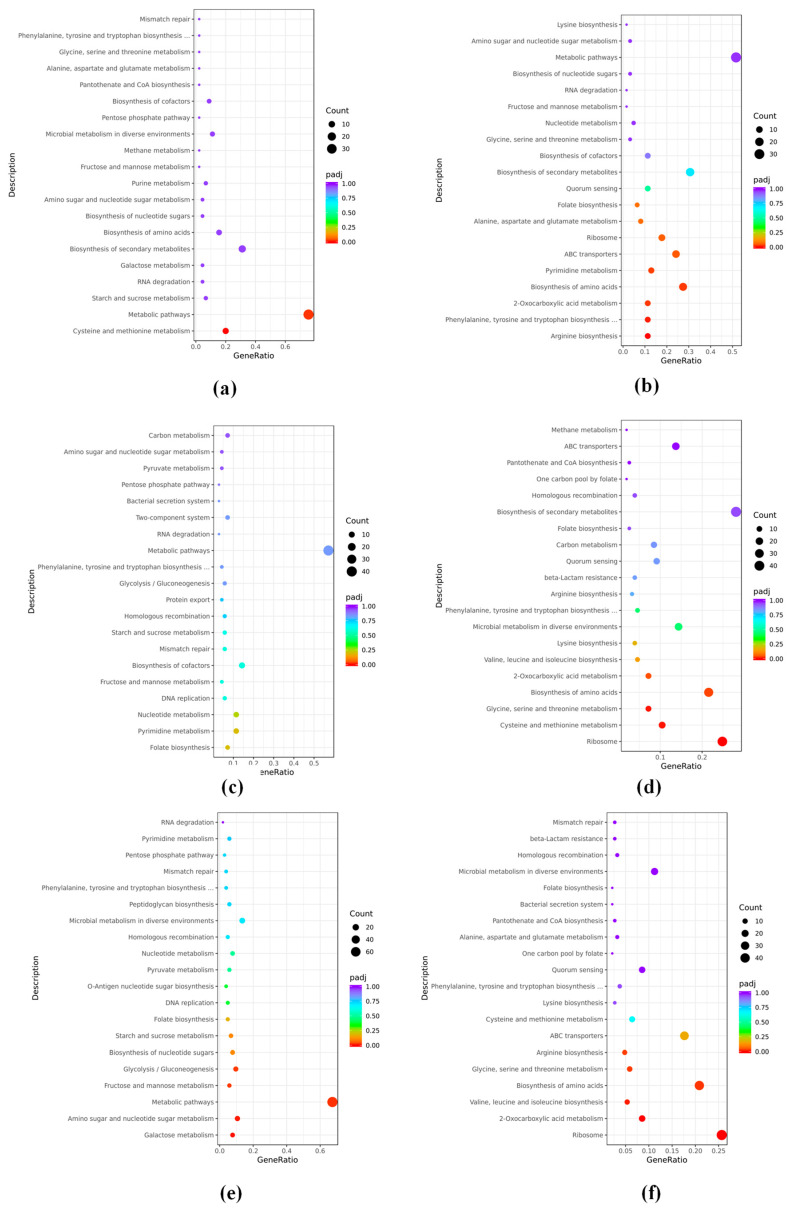
KEGG pathway enrichment in different groups. (**a**,**b**) Snew-2 vs. Snew-1 group enriched up- and down-regulated pathways. (**c**,**d**) Snew-3 vs. Snew-2 group enriched up- and down-regulated pathways. (**e**,**f**) Snew-3 vs. Snew-1 group enriched up- and down-regulated pathways.

In the KEGG pathway enrichment analysis (Figure 6), among the top 20 total enriched pathways in Snew-2 vs. Snew-1, the up-regulated enriched pathways were mainly cysteine and methionine metabolism, metabolic pathways, and starch and sucrose metabolism, and the down-regulated pathways were arginine biosynthesis, Phenylalanine, tyrosine and tryptophan biosynthesis and 2-Oxocarboxylic acid metabolism. At the same time, the down-regulated pathways were arginine biosynthesis, Phenylalanine, tyrosine, and tryptophan biosynthesis, and 2-Oxocarboxylic acid metabolism. Among the top 20 total enriched pathways in Snew-3 vs. Snew-2, the up-regulated pathways were mainly Folate biosynthesis, Pyrimidine metabolism and nucleotide metabolism, biosynthesis of amino acids, Glycine, serine, and threonine metabolism, and cysteine and methionine metabolism. Among the top 20 total enriched pathways in Snew-3 vs. Snew-1, the up-regulated pathways were mainly galactose metabolism, amino sugar and nucleotide sugar metabolism, amino sugar and nucleotide sugar metabolism, and fructose and mannose metabolism, and the down-regulated pathways were mainly 2-Oxocarboxylic acid metabolism, Valine, leucine, and isoleucine biosynthesis, and the biosynthesis of amino acids. Consistent with the differentially expressed genes, starch and sucrose metabolism, galactose metabolism, and the fructose and mannose metabolism pathways of sugar metabolism were all up-regulated enriched pathways. Cysteine and methionine metabolism was up-regulated in the early phase and became down-regulated in the late phase of the enrichment pathway. Aline, leucine, and isoleucine biosynthesis were consistently down-regulated.

### 3.4. Metabolomics Analysis

Based on a one-way ANOVA, all metabolites detected in positive and negative ion modes (including unidentified metabolites) were analyzed for differences. Differential metabolites (Table S2) were screened and categorized using *t*-tests with *p* values < 0.05, VIP values > 1, and |log 2 (Fold Change)| > 0 as cardinal criteria.

#### 3.4.1. Carbohydrates

Table 4 lists the carbohydrates in the three groups of differential metabolites. In the Snew-2 vs. Snew-1 group, there were no significantly up-regulated substances, and the contents of lactose 6-phosphate and Pisatoside were significantly decreasing. In the Snew-3 vs. Snew-2 group, the contents of Maltopentaose and glycogen were increased significantly, while the content of D-Arabinose 5-phosphate was decreasing significantly. In the Snew-3 vs. Snew-1 group, there were three significantly up-regulated substances and eleven significantly down-regulated ones. Fewer substances had significant changes in the first and middle stages of fermentation, and as fermentation progressed, more carbohydrates showed significant changes and more significantly down-regulated substances in the later stages compared to the earlier stages of fermentation.

#### 3.4.2. Proteins and Amino Acids

During Snew fermentation, the number of proteins and amino acids screened was higher than that of carbohydrates, 47 in total (Table 5), and more substances were significantly increased than decreased. In the Snew-2 vs. Snew-1 group, 20 substances, such as (R)-Leucic acid, L-Aspartic acid, and L-tryptophan, were significantly up-regulated, and 11 substances, such as O-acetyl-L-serine, L-Norleucine, and 11 other substances were significantly down-regulated; in the Snew-3 vs. Snew-2 group, fewer substances underwent significant changes. Six substances, such as 4-Aminobutyric Acid and L-threonine, were increased, as well as 3-Methylhistidine, Leu-Leu-OH, and glutamyl glutamic acid; in the Snew-3 vs. Snew-1 group, 23 substances such as D-Proline increased significantly, and 12 substances such as O-acetyl-L-serine decreased significantly. It can be noted that two substances, 4-Aminobutyric Acid and N-γ-L-Glutamyl-D-alanine, were significantly increased in all three groups.

#### 3.4.3. Fats and Fatty Acids

Glycerol 3-phosphate, Malaoxon, Triacetin, and six other substances were significantly increased in the Snew-2 vs. Snew-1 group, while D-Malic acid, γ-Linolenic acid, and four other substances showed a significant decrease. Alpha-Bixin, Millefin, Thromboxane B2, and Ethyl oleate were significantly increased in Snew-3 vs. Snew-2 group. Thromboxane B2 and Ethyl oleate were significantly increased in the Snew-3 vs. Snew-2 group, while Adenosine diphosphate ribose and Rosmarinic acid showed a significant decrease. Cholic acid in the Snew-3 vs. Snew-1 group, Triacetin, and eight other substances were significantly up-regulated, and gamma-Linolenic acid and six other substances were significantly down-regulated in the Snew-3 vs. Snew-1 group (Table 6).

#### 3.4.4. Flavor-Related Substances

For the screened flavor-related substances, again, the number of substances up-regulated was greater than the number of substances down-regulated (Table 7). Nine substances were significantly up-regulated in the Snew-2 vs. Snew-1 group, including 2-Hydroxybutyric acid, L-lactic acid, etc. Further, five substances were significantly down-regulated, such as Ethyl acetoacetate and O-Phosphoethanolamine. In the Snew-3 vs. Snew-2 group, there were four substances with significant changes; 4-Deoxyerythronic acid, Ethyl acetoacetate, and Palmitoylethanolamide were significantly up-regulated, and O-Phosphoethanolamine was significantly down-regulated. In the Snew-3 vs. Snew-1 group, the contents of 10 substances were significantly increased, including Succinic Acid, L-lactic acid, and the contents of six substances were significantly decreased, including Dimethylmalonic acid and 2-Methylbenzoic acid. As a marker substance for fermented milk, L-lactic acid was consistently increased in Snew-fermented milk, except that the change was insignificant in the Snew-3 vs. Snew-2 group.

Significant changes in substance content generally occurred in the first and middle stages of fermentation, with fewer substances showing significant changes in the middle and late stages.

## 4. Discussion

The fermentation process took approximately 7 h to reach a pH of 4.5, and the final TA of the fermented milk was 81.6 °T, which is between the recommended 70–110 °T [34]. Also, the viable bacteria count in Snew-fermented milk was 8.27, which is in line with the national standard and similar to the results of Li et al. [6]. The water holding capacity of Snew-fermented milk was 66.7%, which is higher than the 48.01% in a previous study by Dan et al. [5]. The viscosity at the end of fermentation was 2710 cP, which was better than 615 cP previously determined by Xu et al. [35]. The physicochemical properties of the *S. thermophilus* fermented milk suggest that it can be used as a strain of bacteria to produce fermented milk. The increased content of acidity values, aromatic compounds, ketones, and esters [36], which together give fermented milk its unique flavor, can be used to produce fermented milk with the addition of Snew.

### 4.1. Carbohydrates

**Figure 7:**
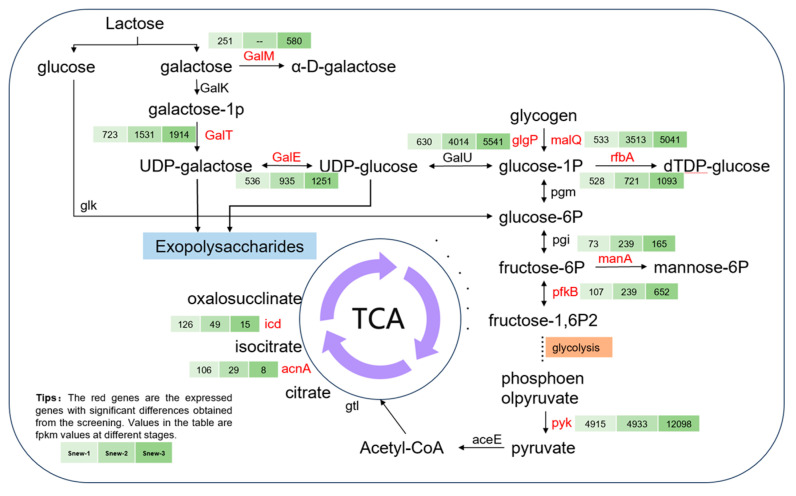
Annotation to expressed genes and metabolites related to carbohydrates.

The joint analysis of transcriptomic and metabolomic results from Snew-fermented milk was conducted (Figure 7). The expression of *malQ* was significantly up-regulated during Snew fermentation. *MalQ* encodes 4-α-glucan transferase, an enzyme that facilitates the conversion of glycogen and starch into α-D-glucose-1-phosphate, thereby promoting the breakdown of sugars within the milk matrix. The gene *manA* encodes mannose-6-phosphate isomerase, enabling the interconversion of mannose-6-phosphate and fructose 6-phosphate. During the fermentation of Snew, the gene expression of *manA* in the late fermentation stage showed an upward trend, while the mannose-6-phosphate content showed a significant decrease in the corresponding stage. Thus, it was hypothesized that Snew completed converting from mannose-6-phosphate to fructose 6-phosphate using mannose-6-phosphate isomerase. Meanwhile, *pfkB* was significantly up-regulated in fermentation, and its encoded phosphofructokinase metabolizes fructose 6-phosphate and participates in the glycolytic pathway related to providing energy for the growth and metabolism of the strain [37]. No significant change in fructose 6-phosphate was shown in the metabolomics results. This may be due to fructose 6-phosphate from mannose-6-phosphate entering pathways such as glycolysis and thus participating in metabolism [38]. *GalM* encodes a galactose-1-conjugating enzyme that converts D-galactose to α-D-galactose. *GalM* expression was significantly up-regulated after fermentation, promoting the production of α-D-galactose and galactose metabolism during fermentation. *GalT* encodes UDP-glucose-hexose-1-phosphate uridylyl transferase, which participates in the process of lactose–galactose metabolism. The expression level of *galT* was significantly elevated during the pre-fermentation stage, which promoted the conversion of α-D-galactose 1-phosphate to α-D-glucose 1-phosphate. The metabolism of galactose 1-phosphate exhibited a decrease in content throughout the fermentation process, which showed a consistent change trend. The *galT* gene is essential and is found in all genomes holding the Leloir pathway, and is not present in any genome without this pathway [39]. A previous study demonstrated that the deletion of *galT* resulted in the accumulation of galactose-1P [40].

### 4.2. Proteins and Amino Acids

**Figure 8:**
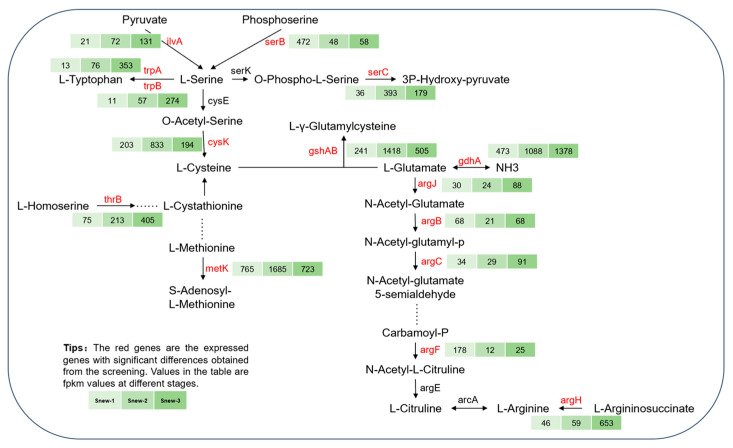
Annotation of expressed genes and metabolites related to proteins and amino acids.

Peptides are hydrolyzed to amino acids by peptidases, and amino acids synthesize flavor substances as precursors [41]. The significant up-regulation of the expression levels of peptidases encoded by *pepC* and *pepA*, as annotated by transcriptomics, and the changes in amino acid content during fermentation, suggest that Snew can also regulate the production of flavor substances through amino acid metabolism. Consistent with Irigoyen [42], who found that the cysteine content increased in fermented milk, in this study, *cysK*-encoded cysteine synthase played an important role in the synthesis of L-cysteine, which was catalyzed by O-acetyl-L-serine to produce L-cysteine. In Snew fermentation (Figure 8)., the expression level of *cysK* increased significantly during the pre-fermentation period, while the decrease in the amount of O-acetyl-L-serine exhibited during the same period further validated the change in *cysK*.

*SerC* encodes phosphoserine aminotransferase, which converts 3P-hydroxy pyruvate to phosphoserine, and its expression was found to be significantly up-regulated in the middle stage of fermentation and down-regulated in the late stage of fermentation. *serB* encodes phosphoserine phosphatase, whose expression was significantly up-regulated after fermentation, which converts phosphoserine to serine. *serB* encodes phosphoserine phosphatase, the expression of which was significantly up-regulated after fermentation, converting phosphoserine to serine, thus revealing that serine synthesis was promoted after Snew fermentation. No significant increase in serine content was found in the metabolites. However, the pyruvate, serylphenylalanine, and serylthreonine content were increased, suggesting the further involvement of serine in pyruvate or other kinds of amino acid metabolism. The results of a previous study also reported a decrease in serine levels [6].

Glutamate plays an important role in organism protein metabolism. *GshAB*, a gene encoding glutamate-cysteine ligase, was significantly up-regulated in the mid-fermentation stage. Its role is to achieve the conversion of cysteine and glutamate to glutamyl cysteine. The metabolites analyzed showed a decrease in glutamate content in the mid-fermentation stage. Meanwhile, *guaA*, a gene encoding glutamine and hydrolyzing GMP synthase, was significantly up-regulated in the late fermentation stage. The metabolism of glutamine to produce ammonia, which was transferred to the ATP-pyrophosphatase structural domain through substrate protection to continue supplying energy for the growth and metabolic process of the strain in the late fermentation stage, further illustrated the decrease in glutamine content after Snew fermentation. *GdhA* and *argJ* encode for glutamate dehydrogenase and glutamate N-acetyltransferase, respectively, and the glutamate content gradually decreases with fermentation. *GdhA* and *argJ* encode for glutamate dehydrogenase and glutamate N-acetyltransferase, respectively, and the glutamate content gradually decreases with fermentation. The expression levels of *gdhA* and *argJ* decreased during the fermentation process, with consistent results.

### 4.3. Fats and Fatty Acids, Flavor-Related Substances

*AccB, accD, fabD, fabF, fabG, fabZ,* and *fabK* encode enzymes related to the fatty acid biosynthesis pathway, and the expression levels of the above genes showed a tendency to increase and then decrease during the fermentation process. Octanoic acid, Decanoic acid, Tetradecanoic acid, and volatile fatty acids such as Hexadecanoic acid and Octadecanoic acid increased significantly in the late stage of fermentation after accumulation in the pre-fermentation stage. Changes in volatile fatty acid content were consistent with Beal’s observation of the fatty acid production content during the fermentation of skimmed milk [43]. The production of these fatty acids promotes the formation of volatile amino acids that confer rich flavor characteristics to fermented milk.

After Snew fermentation, the expression level of *H0506_RS05880* encoding L-lactate dehydrogenase was increased, which promoted the production of lactic acid and was consistent with the results of the increase in the content of L-lactic acid. Increased expression levels of *adhE* encoding alcohol dehydrogenase and *H0506_RS04510* encoding a protein containing the catalytic structural domain of alcohol dehydrogenase promoted the metabolism of alcohols during fermentation. They favored the production of aldehydes and esters. In the analysis of volatile metabolites, it was found that aldehydes and esters increased in the later stages of fermentation, with results that were consistent with the increase in the amount of L-lactic acid. This trend is in agreement with Jia et al. [44], who reported an increase in aldehydes and esters after *S. thermophilus* fermentation. However, they also reported an increase in ketones, alcohols, and terpenoids, which may be due to the influence of factors such as different fermentation substrates and fermentation temperature.

## 5. Conclusions

*S. thermophilus* Snew showed excellent physicochemical properties and metabolic characteristics during the fermentation of cow’s milk, proving its potential as a strain for fermented milk production. The fermentation time of *S. thermophilus* Snew was 7 h. The titratable acidity of Snew-fermented milk was 81.7 °T, and the viable bacterial counts increased and then decreased, while the water holding capacity and viscosity increased all the time. This provides strong support for its use as a fermented milk production strain. From the results of transcriptomic and metabolomic analyses, there were significant differences in the expression of several key genes during the fermentation of Snew, especially those related to sugar metabolism and synthesis, such as *malQ, manA, pfkB, galM, galT*, and so on. The expression of these genes regulates the conversion of sugars, galactose, and other substances in the milk matrix, providing an important basis for the formation of fermented milk texture. In addition, Snew’s peptidase genes (*pepC, pepA*) and amino acid synthesis-related genes (e.g., *cysK, serC, serB*) were significantly up-regulated, which further regulated the production of flavoring substances, especially the metabolism of cysteine and serine, both of which contribute to flavor richness. In terms of volatile compounds, the content of aldehydes, esters, and fatty acids was significantly increased in Snew-fermented milk, and the formation of these compounds is an important reflection of the metabolic activity of Snew and imparts a unique flavor profile to the fermented milk. In particular, the increase in alcohols and esters was closely related to the metabolic pathways of the Snew strain, confirming its role in promoting volatile substances during fermentation. In the future, a deeper connection could be made to the function of fermented milk. This study provides theoretical support for the discovery of *S. thermophilus* and the industrial production of fermented milk.

## Figures and Tables

**Table 1:** Carbohydrate-related differentially expressed genes.

Gene Name	log2 Fold Change	*p* Value	padj
Snew-2 vs. Snew-1			
*malQ*	2.718341772	4.6 × 10^−177^	5.88 × 10^−175^
*glgP*	2.668609544	2.99 × 10^−176^	3.44 × 10^−174^
*galT*	1.078783655	1.75 × 10^−37^	3.65 × 10^−36^
*pfkB*	1.156063005	3.12 × 10^−12^	1.83 × 10^−11^
*acnA*	−1.908925969	1.74 × 10^−54^	5.67 × 10^−53^
*icd*	−1.376243406	1.12 × 10^−21^	1.21 × 10^−20^
Snew-3 vs. Snew-2			
*pyk*	1.30229497	2.38 × 10^−50^	3.01 × 10^−49^
*manA*	1.354145616	2.49 × 10^−16^	1.02 × 10^−15^
*pfkB*	1.451162952	4.65 × 10^−15^	1.77 × 10^−14^
*acnA*	−1.787981157	2.34 × 10^−17^	1.02 × 10^−16^
*leuB*	−1.127073848	9.78 × 10^−11^	2.92 × 10^−10^
*icd*	−1.657133567	3.09 × 10^−10^	8.93 × 10^−10^
Snew-3 vs. Snew-1			
*malQ*	3.213654703	3.25 × 10^−174^	2.81 × 10^−172^
*glgP*	3.107764906	9.03 × 10^−148^	5.20 × 10^−146^
*galT*	1.373909205	1.26 × 10^−52^	1.20 × 10^−51^
*galE*	1.191262155	6.78 × 10^−42^	4.76 × 10^−41^
*rpe*	1.264203236	1.06 × 10^−33^	6.00 × 10^−33^
*pyk*	1.270087216	1.53 × 10^−33^	8.68 × 10^−33^
*pfkB*	2.573492775	3.48 × 10^−30^	1.78 × 10^−29^
*yidA*	1.371341311	7.46 × 10^−26^	3.36 × 10^−25^
*pflA*	1.634802362	8.65 × 10^−25^	3.81 × 10^−24^
*rfbA*	1.020449403	1.06 × 10^−22^	4.41 × 10^−22^
*galM*	1.177938624	7.15 × 10^−16^	2.31 × 10^−15^
*manA*	1.155910505	1.63 × 10^−12^	4.59 × 10^−12^
*acnA*	−3.731728314	7.43 × 10^−103^	2.17 × 10^−101^
*icd*	−3.064991837	3.30 × 10^−46^	2.64 × 10^−45^
*leuB*	−1.710315051	9.29 × 10^−18^	3.28 × 10^−17^
*leuC*	−1.763371819	1.51 × 10^−12^	4.26 × 10^−12^

**Table 2:** Protein and amino acid related differentially expressed genes.

Gene_Name	log2 Fold Change	*p* Value	padj
Snew-2 vs. Snew-1			
*cysK*	2.184038289	1.79 × 10^−97^	1.07 × 10^−95^
*gshAB*	1.48546813	3.34 × 10^−97^	1.48 × 10^−72^
*metK*	1.217993942	4.69 × 10^−35^	8.81 × 10^−34^
*pepC*	1.054827138	8.42 × 10^−28^	1.17 × 10^−26^
*serC*	1.132340201	1.05 × 10^−25^	1.33 × 10^−24^
*argH*	−3.463753221	2.14 × 10^−242^	6.17 × 10^−240^
*argF*	−3.873696562	1.24 × 10^−89^	6.32 × 10^−88^
*trpA*	−2.211582291	2.68 × 10^−53^	8.11 × 10^−52^
*trpB*	−2.259641866	8.51 × 10^−43^	2.02 × 10^−41^
*argC*	−1.675873375	6.25 × 10^−17^	5.00 × 10^−16^
*argJ*	−1.883883431	8.22 × 10^−16^	6.20 × 10^−15^
*argB*	−1.7103422	6.38 × 10^−12^	3.60 × 10^−11^
Snew-3 vs. Snew-2			
*racE*	2.441167534	8.40 × 10^−131^	4.64 × 10^−129^
*dnaK*	1.644091756	9.57 × 10^−89^	3.24 × 10^−87^
*def*	1.989963937	8.84 × 10^−74^	1.88 × 10^−72^
*guaA*	1.29999676	3.27 × 10^−44^	3.37 × 10^−43^
*msrB*	1.448785684	4.36 × 10^−34^	3.44 × 10^−33^
*hprK*	1.361945345	2.55 × 10^−25^	1.48 × 10^−24^
*argF*	1.051127935	0.000803917	0.001385544
*gshAB*	−2.550430051	1.24 × 10^−183^	1.37 × 10^−181^
*cysK*	−2.113828163	1.52 × 10^−71^	3.12 × 10^−70^
*gdhA*	−1.195338163	2.04 × 10^−26^	1.23 × 10^−25^
*thrB*	−1.499101259	1.70 × 10^−25^	9.95 × 10^−25^
*metK*	−1.13041765	8.07 × 10^−25^	4.60 × 10^−24^
*trpA*	−2.591031854	8.99 × 10^−21^	4.56 × 10^−20^
*trpB*	−2.316529102	8.11 × 10^−19^	3.85 × 10^−18^
*ilvA*	−1.774912883	3.28 × 10^−16^	1.32 × 10^−15^
*serC*	−3.429518071	2.16E × 10^−117^	1.09 × 10^−115^
*serB*	3.299953042	1.46 × 10^−60^	2.43 × 10^−59^
Snew-3 vs. Snew-1			
*dnaK*	4.253878659	0	0
*racE*	2.016661758	6.98 × 10^−94^	1.77 × 10^−92^
*msrB*	2.337439758	6.77 × 10^−73^	1.15 × 10^−71^
*def*	1.822626872	2.11 × 10^−42^	1.52 × 10^−41^
*pepA*	1.412777226	2.39 × 10^−37^	1.48 × 10^−36^
*murD*	1.283893541	2.53 × 10^−30^	1.31 × 10^−29^
*hprK*	1.251139811	3.83 × 10^−18^	1.36 × 10^−17^
*argH*	−3.849423215	5.28 × 10^−211^	7.01 × 10^−209^
*trpA*	−4.837336435	1.69 × 10^−92^	3.95 × 10^−91^
*trpB*	−4.611182172	1.69 × 10^−69^	2.50 × 10^−68^
*thrB*	−2.463745464	2.31 × 10^−68^	3.32 × 10^−67^
*argF*	−2.857706259	2.02 × 10^−54^	2.03 × 10^−53^
*ilvA*	−2.674528569	9.58 × 10^−48^	8.11 × 10^−47^
*serC*	−2.332518097	5.27 × 10^−47^	4.32 × 10^−46^
*gdhA*	−1.572806515	1.17 × 10^−43^	8.71 × 10^−43^
*gshAB*	−1.099615019	4.52 × 10^−30^	2.31 × 10^−29^
*asnA*	−1.114000874	4.48 × 10^−27^	2.08 × 10^−26^
*argC*	−1.438985351	1.93 × 10^−14^	5.86 × 10^−14^
*tatC*	−1.365169765	4.96 × 10^−11^	1.32 × 10^−10^
*argJ*	−1.567432357	9.23 × 10^−10^	2.31 × 10^−09^
*serB*	2.991286395	6.59 × 10^−46^	5.25 × 10^−45^

**Table 3:** Fat and fatty acid-related differentially expressed genes.

Gene_Name	log2 Fold Change	*p* Value	padj
Snew-2 vs. Snew-1			
*trpC*	−2.002318	1.85 × 10^−32^	3.20 × 10^−31^
Snew-3 vs. Snew-2			
*birA*	1.1057081	8.45 × 10^−19^	3.98 × 10^−18^
*gap*	1.0810631	4.77 × 10^−42^	4.57 × 10^−41^
Snew-3 vs. Snew-1			
*gap*	1.3875187	4.29 × 10^−41^	2.89 × 10^−40^

**Table 4:** Carbohydrate differential metabolites. ‘——’ means not eligible for screening.

Serial Number	MS2 Name	mz	Type	HMDB	CAS	Log_Foldchange (Snew-2 vs. Snew-1)	Log_Foldchange (Snew-3 vs. Snew-2)	Log_Foldchange (Snew-3 vs. Snew-1)
1	2-Phospho-D-glyceric acid	184.9856944	NEG	HMDB0003391		——	——	−3.152304111
2	Galactose 1-phosphate	259.0221309	NEG	HMDB0000645	2255-14-3	——	——	−1.657307301
3	UDP-N-acetyl-alpha-D-galactosamine	606.0748035	NEG	HMDB0060522		−1.643705856	——	−1.278777455
4	Mannose-6-phosphate	283.018994	POS	HMDB0001078	3672-5-9	——	——	−1.452997144
5	D-Arabinose 5-phosphate	229.0116641	NEG	HMDB0011734	13137-52-5	——	−1.151936341	−1.732634817
6	D-Xylitol	151.061181	NEG	HMDB0002917	87-99-0	——	——	−2.528827239
7	L-Gulose	179.0559985	NEG	HMDB0012326	6027-89-0	——	——	1.376313123
8	Lactose 6-phosphate	423.0895144	POS	HMDB0006789		−1.836706702		−2.480846476
9	Maltopentaose	827.2675388	NEG	HMDB0012254	34620-76-3	——	2.631605776	1.717822319
10	Aminofructose 6-phosphate	260.0527268	POS	HMDB0060436		——	——	−1.433177881
11	Pisatoside	262.0919824	POS	HMDB0039127	18814-39-6	−1.412839842	——	——
12	Lacto-N-biose I	384.1493767	POS	HMDB0006575	50787-09-2	−1.143885106	——	−1.90190282
13	Fructose 1-phosphate	283.0187485	POS	HMDB0001076	15978-08-2	——	——	−2.07611233
14	Glycogen	667.2284548	POS	HMDB0000757	9005-79-2	——	1.421856058	1.275051114
15	Isobiflorin 6’‘-gallate	507.1017752	POS	HMDB0040633	152041-17-3	−2.679094398	——	−2.534443751

**Table 5:** Protein and amino acid differential metabolites. ‘——’ means not eligible for screening.

Serial Number	MS2 Name	mz	Type	HMDB	CAS	Log_Foldchange (Snew-2 vs. Snew-1)	Log_Foldchange (Snew-3 vs. Snew-2)	Log_Foldchange (Snew-3 vs. Snew-1)
1	D-Proline	116.0703814	POS	HMDB0003411	344-25-2	——	——	1.239156434
2	Glycylproline	173.0917953	POS	HMDB0000721	704-15-4	——	——	1.035647091
3	3-Methylhistidine	170.0918807	POS	HMDB0000479	368-16-1	——	−1.30026032	——
4	(R)-Leucic acid	131.0712502	NEG			1.42910805	——	2.37194096
5	Threoninyl-Proline	217.1180678	POS	HMDB0029069		1.479765893	——	1.936918789
6	N-Acryloylglycine	130.0496469	POS	HMDB0001843	24599-25-5	——	——	−1.239882619
7	L-Aspartic acid	134.0445675	POS	HMDB0000191	56-84-8	2.641157455	——	3.464294585
8	O-Acetyl-L-serine	148.0601266	POS			−1.570118631	——	−1.596467587
9	Racemethionine	150.0581111	POS			−5.446794224	——	−4.002776366
10	L-Norleucine	132.1017631	POS	HMDB0001645	327-57-1	−1.375945356	——	——
12	4-Aminobutyric Acid	86.05986947	POS			3.505695857	1.744559379	5.250255236
13	Leu-Leu-OH	245.1854514	POS			——	−2.025758795	−2.54325273
14	L-Glutamic acid	148.0604875	POS	HMDB0000148	56-86-0	−1.155432813	——	——
15	5-Aminopentanamide	117.101863	POS	HMDB0012176	13023-70-6	1.465592532	——	1.300286522
16	L-Tyrosine	182.0809316	POS	HMDB0000158	60-18-4	−1.130292372	——	——
17	D-Glutamine	147.0764651	POS	HMDB0003423	5959-95-5	——	——	−1.077047271
18	Leucyl-Valine	231.1700626	POS	HMDB0028942		1.341278426	——	1.425630639
19	D-4’-Phosphopantothenate	300.083964	POS	HMDB0001016		——	——	1.57956457
20	Serylphenylalanine	253.1177193	POS	HMDB0029046		1.392284337	——	——
21	Valyl-Aspartate	233.1129817	POS	HMDB0029123		1.738274256	——	1.481879761
22	L-Threonine	118.0509202	NEG	HMDB0000167	72-19-5	——	1.155982078	1.405676737
23	L-Tryptophan	203.0823289	NEG	HMDB0000929	73-22-3	1.736889068	——	2.139601727
24	N-methyl-L-glutamic Acid	160.061294	NEG			——	1.093118525	1.629872985
25	(+)-threo-2-Amino-3,4-dihydroxybutanoic acid	136.0615295	POS	HMDB0029389	21768-44-5	——	1.24012642	2.017581484
26	N-Acetyl-L-methionine	190.0543367	NEG	HMDB0011745	65-82-7	1.956883451	——	——
27	Leucyl-Tryptophan	318.1805253	POS	HMDB0028940		−2.969584112	2.142119924	——
28	N-gamma-L-Glutamyl-D-alanine	219.0972013	POS	HMDB0036301		1.643911623	1.046254414	2.690166037
29	Glutamylglutamic acid	275.0885159	NEG	HMDB0028818	3929-61-1	1.188033016	−3.087835061	−1.899802045
30	Serylthreonine	207.0970272	POS	HMDB0029049		1.707408463	——	1.908408873
31	N-Ribosylhistidine	288.1193122	POS	HMDB0002089	98379-91-0	3.738475435	——	4.378293801
32	Aminoadipic acid	162.0759372	POS	HMDB0000510	542-32-5	——	——	1.944654843
33	Lycoperdic acid	218.0658825	POS	HMDB0030417	69086-72-2	1.456605936	——	1.24166744
34	N-Acetyl-L-glutamate 5-semialdehyde	174.0756396	POS	HMDB0006488	13074-21-0	−1.909457996	——	−2.619748286
35	DL-Dopa	196.0542732	NEG	HMDB0000609	63-84-3	——	——	−1.114802981
36	Glutamyllysine	276.1551211	POS	HMDB0004207	5891-53-2	1.327179339	——	1.128827514
37	alpha-Fluoro-beta-alanine	108.0473355	POS	HMDB0060434		1.02014938	——	——
38	N-Acetylglutamic acid	190.070578	POS	HMDB0001138	1188-37-0	−1.778283797	——	−1.733471511
39	Glutamylvaline	247.1288181	POS	HMDB0028832	1453453	1.194894677	——	——
40	N-Acetyl-L-phenylalanine	206.0820543	NEG	HMDB0000512	2018-61-3	−1.079576443	——	——
41	Pyrroline hydroxycarboxylic acid	130.0496064	POS	HMDB0001369	22573-88-2	−1.910622708	——	−2.744749749
42	Pyroglutamic acid	128.0352063	NEG	HMDB0000267	98-79-3	1.396960546	——	1.607474316
43	L-phenylalanyl-L-hydroxyproline	279.1311367	POS	HMDB0011176		——	——	1.108318363
44	(1R)-Hydroxy-(2R)-glutathionyl-1,2-dihydronaphthalene	452.139838	POS	HMDB0060300		1.028078485	——	1.160302281
45	Enalaprilat	385.1967185	POS	HMDB0041886	76420-72-9	−7.274947703	——	−7.265053096
46	L-Lysine	145.0983025	NEG	HMDB0000182	56-87-1	——	——	−1.519368884
47	Arginyl-Glutamine	303.1700908	POS	HMDB0028707		3.258171656	——	——

**Table 6:** Fat and fatty acid differential metabolites. ‘——’ means not eligible for screening.

Serial Number	MS2 Name	mz	Type	HMDB	CAS	Log_Foldchange (Snew-2 vs. Snew-1)	Log_Foldchange (Snew-3 vs. Snew-2)	Log_Foldchange (Snew-3 vs. Snew-1)
1	D-Malic acid	133.0141159	NEG	HMDB0031518	636-61-3	−1.673843572	——	−2.071851733
2	Gaultherin	491.1509312	NEG			1.877593117	——	——
3	Glycerol 3-phosphate	173.0207842	POS	HMDB0000126	57-03-4	1.618910068	——	——
4	Cholic acid	426.3217102	POS	HMDB0000619	81-25-4	——	——	1.180327084
5	Malaoxon	313.0443193	NEG	HMDB0060627		1.869420702	——	1.741542683
6	2-acetyl-1-alkyl-sn-glycero-3-phosphocholine	524.3697249	POS	HMDB0062195	74389-68-7	−1.484277824	——	−1.511442608
7	Adenosine diphosphate ribose	558.0635432	NEG	HMDB0001178	20762-30-5	——	−1.020493396	——
8	alpha-Bixin	393.2134754	NEG	HMDB0035317	6983-79-5	——	1.851305868	1.308075625
9	Acetoacetyl-CoA	850.1210582	NEG	HMDB0001484	1420-36-6	——	——	−1.25725177
10	3-O-fucopyranosyl-2-acetamido-2-deoxyglucopyranose	368.1426577	POS	HMDB0006700	52630-68-9	——	——	−1.341673126
11	2,4,6-Octatriynoic acid	133.0314219	POS	HMDB0030967	51193-80-7	1.663503376	——	——
12	Millefin	351.1760588	POS	HMDB0036689	39262-27-6	——	1.687214603	1.486912271
13	Thromboxane B2	393.2234785	POS	HMDB0003252	54397-85-2	——	2.033551659	2.822698113
14	gamma-Linolenic acid	277.2168922	NEG			−1.623749773	——	−1.21526795
15	Rosmarinic acid	359.0745372	NEG	HMDB0003572	537-15-5	——	−1.585508348	——
16	7-Epi-12-hydroxyjasmonic acid glucoside	389.1731302	POS	HMDB0040706	124649-25-8	1.33647295	——	——
17	Methyl helianthenoate F glucoside	355.1342943	POS	HMDB0040797		——	——	1.025778003
18	12-Methyltridecanoic acid	227.2014079	NEG	HMDB0031072	2724-57-4	−1.241158351	——	−1.68142511
19	Ethyl oleate	309.2797545	NEG	HMDB0034451	111-62-6	——	1.524972271	1.954656169
20	Triacetin	219.0952323	POS	HMDB0029592	102-76-1	1.406441601	——	1.262812126

**Table 7:** Flavor-related class of differential metabolites. ‘——’ means not eligible for screening.

Serial Number	MS2 Name	mz	Type	HMDB	CAS	Log_Foldchange (Snew-2 vs. Snew-1)	Log_Foldchange (Snew-3 vs. Snew-2)	Log_Foldchange (Snew-3 vs Snew-1)
1	Succinic Acid	117.019259	NEG			——	——	1.23404739
2	2-Hydroxybutyric acid	103.0399519	NEG	HMDB0000008	600-15-7	3.752027473	——	——
3	Formylanthranilic acid	164.0354262	NEG	HMDB0004089	3342-77-6	2.011638301	——	2.371591562
4	L-Lactic acid	89.02430316	NEG	HMDB0000190	79-33-4	1.612536327	——	2.187635502
5	Indolelactic acid	204.066557	NEG	HMDB0000671	1821-52-9	2.58370578	——	——
6	DL-3-Phenyllactic acid	165.0557941	NEG			2.514627022	——	2.489940596
7	4-Deoxyerythronic acid	87.04381311	POS	HMDB0000498	759-06-8	——	1.717605712	1.840751604
8	Benzoic acid	121.0293922	NEG	HMDB0001870	65-85-0	1.555592096	——	2.036861899
9	Pyruvic acid	87.00872587	NEG	HMDB0000243	127-17-3	1.01546302	——	1.22872031
10	β-Hydroxyisovaleric acid	117.0556415	NEG			2.534231969	——	2.849942233
11	Ethyl acetoacetate	129.0557303	NEG	HMDB0031216	141-97-9	−4.036988605	3.443989081	——
12	Dimethylmalonic acid	131.0350031	NEG	HMDB0002001	595-46-0	——	——	−1.92328009
13	Clarycet	201.1480483	POS	HMDB0036029	131766-73-9	−1.841524016	——	−2.794665986
14	2-Methylbenzoic acid	135.0451507	NEG	HMDB0002340	118-90-1	——	——	−1.21000802
15	Palmitoylethanolamide	300.2888586	POS	HMDB0002100	544-31-0	——	1.238409623	1.10304278
16	Quininic acid	202.0508066	NEG			−3.302771806	——	−2.513653389
17	2,6-Dimethoxybenzoic acid	181.0504004	NEG	HMDB0029273	1466-76-8	3.612863685	——	4.331433024
18	O-Phosphoethanolamine	140.0102044	NEG	HMDB0000224	1071-23-4	−1.089724191	−2.293180533	−3.382904723
19	[4]-Gingerdiol 3,5-diacetate	351.1765767	NEG	HMDB0039132	53254-50-5	−1.822314956	——	−2.39972437

## Data Availability

The original contributions presented in this study are included in the article/Supplementary Materials. Further inquiries can be directed to the corresponding authors.

## Supplementary Materials

The following supporting information can be downloaded at: https://www.mdpi.com/article/10.3390/foods14030530/s1, Table S1: Quality assessment of sample raw sequencing data; Table S2: Differential metabolites; Table S3: Differentially expressed genes; Table S4: Transcriptomics raw data. Table S5: Corresponding parameters of the sensor.
